# Cervical chylous leakage following esophagectomy that was successfully treated by intranodal lipiodol lymphangiography: a case report

**DOI:** 10.1186/s12893-017-0218-x

**Published:** 2017-02-28

**Authors:** Tatsuro Tamura, Naoshi Kubo, Akira Yamamoto, Katsunobu Sakurai, Takahiro Toyokawa, Hiroaki Tanaka, Kazuya Muguruma, Masakazu Yashiro, Kiyoshi Maeda, Kosei Hirakawa, Masaichi Ohira

**Affiliations:** 1 Department of Surgical Oncology, Osaka City University, Graduate School of Medicine, 1-4-3 Asahi-machi, Abeno-ku, Osaka, Japan; 2 Departments of Gastroenterological Surgery, Osaka City General Hospital, 2-13-22 Miyakojima Hondori Miyakojimaku, Osaka, 534-0021 Japan; 3 Department of Diagnostic and Interventional Radiology, Osaka City University, Graduate School of Medicine, 1-4-3 Asahi-machi, Abeno-ku, Osaka, Japan

**Keywords:** Esophageal cancer, Cervical chylous leakage, Lipiodol lymphangiography

## Abstract

**Background:**

Chylous leakage is a well-known complication after esophagectomy, but cervical chylous leakage is relatively rare, and considerable controversy remains regarding the appropriate management strategies. We herein report a case of cervical chylous leakage treated successfully by lipiodol lymphangiography.

**Case presentation:**

The patient, a 70-year-old man with middle thoracic esophageal cancer, underwent radical esophagectomy with 3-field lymph node dissection and subsequently developed cervical chylous leakage. From the second postoperative day (POD2), the amount of fluid in the cervical drainage tube increased by 200–300 ml/day. We started octreotide (300 μg/day) on POD5 and etilefrine (120 mg/day) on the POD6. However, the amount of cervical discharge did not decrease. We performed lipiodol lymphangiography on POD8. Thereafter, the amount of cervical discharge finally began to decrease. We removed the drainage tube on POD13, and the patient was discharged from the hospital on POD23.

**Conclusions:**

Our case suggests the clinical efficacy of lipiodol lymphangiography for cervical chylous leakage after esophagectomy.

## Background

Chylous leakage is a complication that may occur after esophagectomy, as a result of intraoperative injury or inadequate ligation of the thoracic duct, thus leading to chylous leakage. The reported incidence of this complication varies between 1.3 and 3.4% after procedures involving the thorax and neck [1, 2]. Undiagnosed postoperative chylous leakage may lead to malnutrition, sepsis, and a high mortality rate [3, 4]. Therefore, a prompt diagnosis and optimum management are required. Recently, etilefrine and octreotide have been reported to be effective in treating chylous leakage. However, conclusive findings regarding the efficacy of this regimen have yet to be obtained. We herein report a case of esophagectomy complicated by cervical chylous leakage that we treated successfully with lipiodol lymphangiography.

## Case presentation

A 70-year-old man was referred to our hospital for the surgical management of a tumor in the thoracic upper esophagus. A physical examination and laboratory data revealed no remarkable findings. Computed tomography (CT) revealed that the tumor, which was located in the upper thoracic esophagus, had not locally invaded other organs, but there was a metastasis in the right recurrent laryngeal nerve lymph node. Upper gastrointestinal endoscopy showed a 2.5-cm-long ulcerated type 2 tumor in the upper thoracic esophagus. The extent of tumor invasion was estimated to be T2. A histopathological examination of a biopsy specimen revealed the lesion to be squamous cell carcinoma. Pathologic findings showed squamous cell carcinoma of the esophagus, (cT2, N1, M0, cStage II), determined based on the Japanese Classification of Esophageal Cancer, 10th edition. Neoadjuvant chemotherapy consisting of cisplatin and 5-fluorouracil (FP) was performed before surgery. The patient received 2 cycles of the following regimen: intravenous infusions of cisplatin (80 mg/m^2^) on Day 1 and continuous intravenous infusion of 5-fluorouracil (800 mg/m^2^) from Days 1 to 5, every 4 weeks. The tumor and lymph node metastasis were reduced after two cycles, and the neoadjuvant chemotherapy evaluation was deemed a partial response (PR).

The patient underwent subtotal esophagectomy and three-field lymphadenectomy (mediastinal lympadenectomy accompanied with neck and abdominal lymphadenectomy) via thoracoscopy and hand-assisted laparoscopy, and esophagogastrostomy was performed for reconstruction at the neck via the retrosternal route. The thoracic duct was preserved, because the thoracic duct did not approach the tumor and there was no remarkable damage to the thoracic duct during the operation. The histological diagnosis was moderately differentiated squamous cell carcinoma of the esophagus (pT1b, N1, M0, pStage II) based on the Japanese Classification of Esophageal Cancer, 10th edition.

**Fig. 1:**
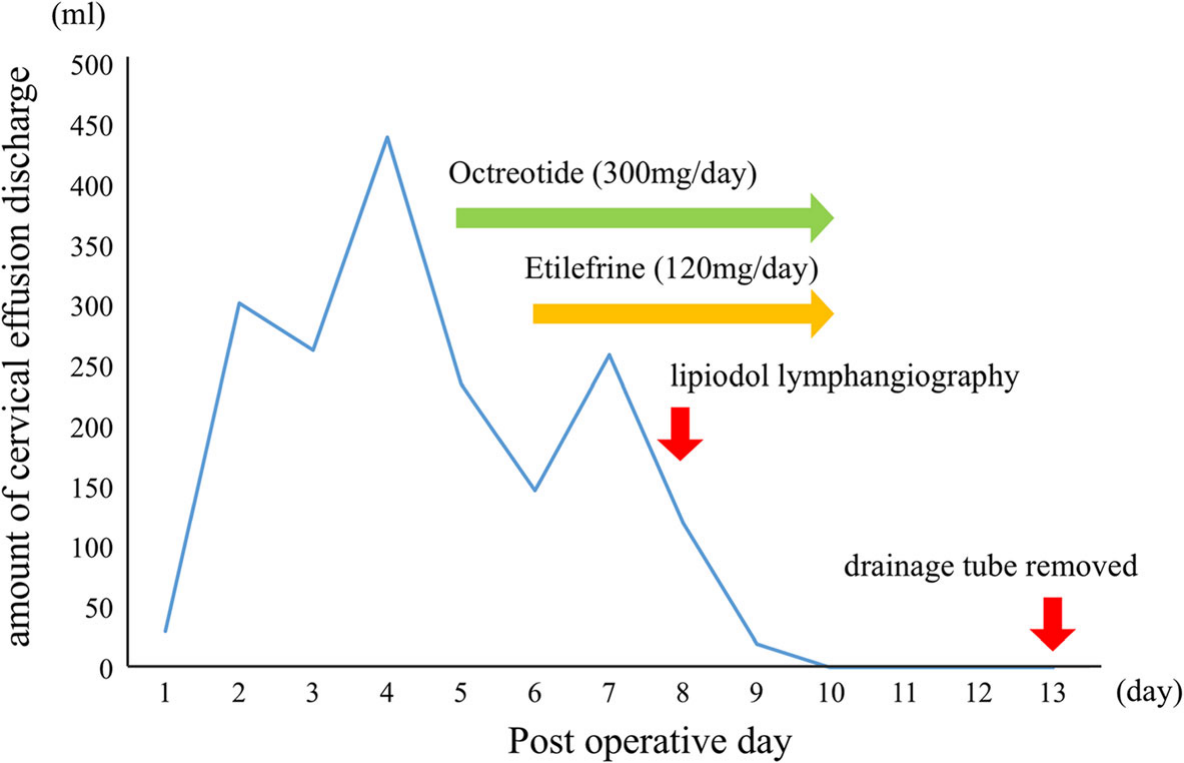
Postoperative progress and amount of discharge from cervical drainage tube

**Fig. 2:**
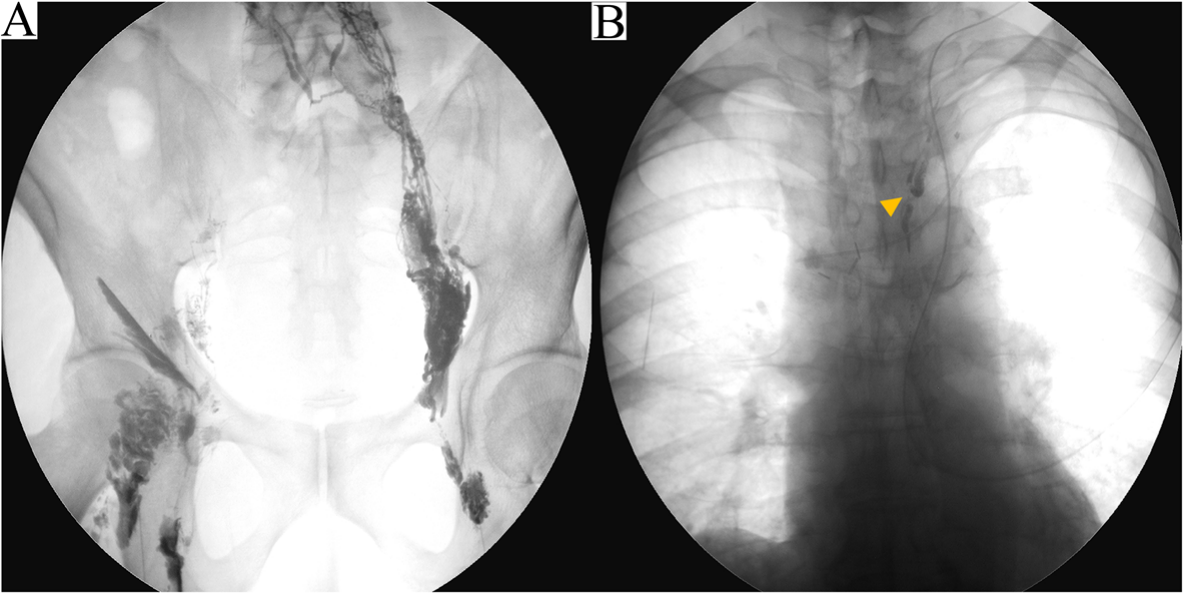
Lipiodol lymphangiography. The injected lipiodol flowed up through the lymph duct via the bilateral inguinal lymph nodes (**a**). A fluoroscopic image obtained after 10 ml of lipiodol was injected shows the accumulation of lipiodol at the ductus thoracicus (*arrowhead*) (**b**)

Postoperatively (Fig. 1), a large amount of cervical effusion discharge (100–450 ml/day) flowed continuously from the cervical drainage tube. From the second postoperative day (POD2), due to the amount of discharge from cervical drainage tube increasing by 200–300 ml/day, we suspected chylous leakage at the neck point and administered an elemental diet (Elental) via an intestinal fistula catheter. But the amount of discharge did not decrease. We started octreotide (300 μg/day) on the POD5 and etilefrine (120 mg/day) on POD6. The amount of discharge decreased only slightly, so we consulted a radiologist at our hospital and performed lipiodol lymphangiography on POD8. The bilateral inguinal lymph nodes were directly accessed under ultrasound guidance with a 23-gauge needle. Lipiodol was manually injected at about 1 ml per 5 min. The lipiodol injection was observed under fluoroscopic guidance in order to confirm appropriate access by the needle (Fig. 2a). After approximately 10 ml of lipiodol had been injected, a fluoroscopic image showed accumulation of the lipiodol at the ductus thoracicus (Fig. 2b).

**Fig. 3:**
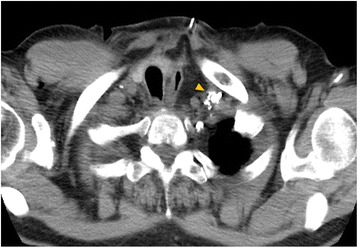
Coronal sections by CT obtained a day after lipiodol lymphangiography show the accumulation of lipiodol at the left venous angle (*arrowhead*)

A day after lymphangiography, CT showed the accumulation of lipiodol at the left venous angle (Fig. 3). After lymphangiography, the amount of discharge from the cervical drainage tube decreased immediately. On POD9 (1 day after lymphangiography), the patient resumed normal enteral nutrition via the intestinal fistula catheter. On POD10 (2 days after lymphangiography), the cervical discharge disappeared. On POD13 (5 days after lymphangiography), the drainage tube was removed. Cervical discharge was not seen at all after the removal of the tube. On POD22 (14 days after lymphangiography), the patient was discharged home.

## Discussion

Postoperative chylous leakage is a rare but well-known complication of general surgery. The reported incidence of this complication varies between 1.3 and 3.4% after procedures involving the thorax and neck [1, 2]. Prophylactic ligation of thoracic duct during the radical resection of esophageal cancer is usually used to prevent and treat chylous leakage. But, there is dispute about prophylactic ligation of the thoracic duct. Fu et al. showed a statistically significant increase in postoperative chylous leakage following ligation, suggesting that prophylactic thoracic ligation was not only unnecessary but also harmful [3]. Basically, we preserve the thoracic duct other than the case that a tumor approaches it.

Chylous leakage can be life-threatening because of the significant loss of fluid, plasma protein, fat, and immunoregulatory lymphocytes, and affected patients exhibit clinical features of severe malnutrition, hyponatremia, acidosis, hypocalcaemia and susceptibility to infection. Therefore, the mortality rate is high in patients with uncontrolled chylous leakage [4, 5]. The initial treatment of chylous leakage is usually conservative therapy, such as fasting, a modified diet (low-fat, medium-chain triglycerides; parenteral nutrition; and supplemental elements), drainage of effusion, and the administration of octreotide [6, 7] and etilefrine [8]. If conservative therapy fails, surgical interventions such as thoracic duct ligation should be considered. However, the thoracic duct and site of chylous leakage are often difficult to locate during reoperation. In addition, because such surgery is considered to increase the risk of complications, great care must be taken during reoperation.

Lipiodol lymphangiography is traditionally a diagnostic tool for identifying chylous leakage, allowing for the detection of the leakage point if surgical intervention is needed [5, 9]. However, lipiodol lymphangiography can also be used as a treatment method itself and not merely to obtain a diagnosis. Even when pleural drainage exceeded 500 ml/day, lipiodol lymphangiography proved an effective treatment in 35% of patients with chylothorax. The successful treatment rate in patients with failed non-surgical treatment was reported to be 51% [10]. Matsumoto et al. reported that 89% patients with postoperative chylous leakage needed no surgical reintervention and that chylous leakage stopped after lipiodol lymphangiography [5]. The mechanism of chylous leakage cessation after lipiodol lymphangiography was suspected to involve the accumulation of lipiodol at the leakage point, inducing an inflammatory reaction and acting as an embolic agent [10].

The traditional procedure of lymphangiography involves the subcutaneous injection of an oily contrast medium, such as lipiodol, into each foot. The traditional procedure has some problems. First by, it is an invasive procedure requiring dorsal incisions, so that it has the potential to lead infection associated with the incisions. Secondly, it is technically challenging because it requires the isolation and cannulation of the fine pedal lymphatic vessels. Even after successful placement of lymphangiogram needles, minimal movement of the patient can dislodge the needles. Whereas, Intranodal lymphangiography using ultrasound is a less-invasive approach in terms of requiring no incision compared to the traditional procedure. Intranodal lymphangiography is simpler and easier because an inguinal node is directly accessed under ultrasound guidance with a 23- gauge to 26-gauge spinal needle with use of a local anesthesia [11, 12].

We performed lipiodol lymphangiography using ultrasound-guided bilateral inguinal lymph node puncture in our patient with cervical chylous leakage following esophagectomy and successfully treated his cervical chylous leakage. Although there are several reports describing lipiodol lymphangiography as effective against thoracic chylous leakage, few have described the effective treatment of cervical chylous leakage using this technique. In the otolaryngology region, Neel et al. reported a case that was treated against the cervical chylous leakage after laryngectomy with bilateral selective neck dissection using bilateral pedal lymphangiography with iodinated oily contrast medium [13]. But there are few other reports that were treated with lymphangiography, there are many reports treated with thoracic duct ligation for cervical chylous leakage in after the head and neck surgery. Our case demonstrated that lipiodol lymphangiography using ultrasound-guided bilateral inguinal lymph node puncture can indeed be an effective conservative treatment not only for thoracic chylous leakage, but also for cervical chylous leakage.

The limitations of a single case report must be acknowledged; although the proposed benefits of lipiodol lymphangiography by bilateral inguinal lymph node puncture may indeed increase the utilization of thoracic duct embolization, firm conclusions regarding its safety and efficacy require further investigation in a prospective series.

Cervical chylous leakage is relatively rare, and considerable controversy remains regarding the appropriate management strategies. Our case suggests the clinical efficacy of lipiodol lymphangiography for cervical chylous leakage after esophagectomy.
